# Preclinical evidence of ghrelin as a therapeutic target in epilepsy

**DOI:** 10.18632/oncotarget.18349

**Published:** 2017-06-02

**Authors:** Tongtong Ge, Wei Yang, Jie Fan, Bingjin Li

**Affiliations:** ^1^ Jilin Provincial Key Laboratory on Molecular and Chemical Genetics, The Second Hospital of Jilin University, Changchun 130041, PR China

**Keywords:** ghrelin, epilepsy, hippocampus, memory, neuroprotection

## Abstract

Ghrelin, an orexigenic peptide synthesized by endocrine cells of the gastric mucosa, plays a major role in inhibiting seizures. However, the underlying mechanism of ghrelin's anticonvulsant action is still unclear. Nowadays, there are considerable evidences showing that ghrelin is implicated in various neurophysiological processes, including learning and memory, neuroprotection, neurogenesis, and inflammatory effects. In this review, we will summarize the effects of ghrelin on epilepsy. It may provide a comprehensive picture of the role of ghrelin in epilepsy.

## INTRODUCTION

Epilepsy is one of the most common chronic neurological disorders. There are approximately more than seventy million people in the world affected by epilepsy [1, 2]. It is reported that approximately 30% patients with epilepsy are thought to have drug-resistant seizures [3]. Human epilepsies belong to a group of disorders characterized by recurrent self-sustained paroxysmal changes in neurologic function. The seizures are caused by excessive electrical discharges in a specific group of neurons due to the demolition of the delicate balance between depression and excitation in the brain [4, 5]. The two key neurotransmitters, the inhibitory transmitter gamma-aminobutyric acid (GABA) and the excitatory transmitter glutamate, play a major role in the misbalance [6].

The search for new antiepileptic drug (AED) therapy is sustained developed [7]. Nevertheless, one third of epileptic patients still have seizures and their epilepsies are resistant to all currently available treatments [8]. Antiepileptic drugs (AEDs) can exert therapeutic effects via modulating the second messengers and neurotransmission or targeting ion channels or ionotropic receptors [9, 10]. However, current AEDs treatments are reported to interfere in the learning, cognition and behavior due to their inferior binding affinity and potency for their receptors [11, 12]. In recent years, more and more studies focus on the relationship between epilepsy and the neuroendocrine regulation. A number of neuropeptides have been implicated with the pathogenesis of epilepsy [6]. Several studies demonstrated that ghrelin may play a significant role in epilepsy. Exogenous ghrelin inhibits the development and severity of PTZ-induced seizures in rats [13]. However, the present clinical studies about the relationship of ghrelin and epilepsy are contradictory due to variable factors. There's a dearth of research on the underlying cellular and molecular mechanisms of ghrelin's antiepileptic actions. In this article, we present a review to introduce the effects of ghrelin on epilepsy and illuminate ghrelin's advantage of being a therapeutic target in epilepsy.

### Ghrelin and its receptors

Ghrelin is a multifunctional 28-amino acid hormone [14], which is known as the endogenous ligand of the synthetic growth hormone secretagogue receptor 1a (GHS-R) ligands [15]. Ghrelin is mainly produced by enteroendocrine X/A-like cells of stomach [16, 17]. Ghrelin gene was conserved throughout the evolution sharing 82.9% homology between rodents and human [18]. In humans, the ghrelin gene is located in chromosome 3p25-26, and the main messenger RNA (mRNA) codifies ghrelin and several bioactive molecules, including des-acyl ghrelin and obestatin [19, 20].

There are two forms of circulating ghrelin, including acylated ghrelin and des-acyl ghrelin [21]. Ghrelin is characterized by the presence of an n-octanoylation (acylated) on the hydroxy group of the third residue, a serine (Ser3), by ghrelin-O-acyltransferase (GOAT) [18]. This post-transcriptional modification is indispensable for binding to GHS-R. It is used to be confirmed that ghrelin is biologically active only in acylated form [14]. However, des-acyl represents approximately 80–90% of the total ghrelin detected in the serum, whether or not des-acyl ghrelin represents a precursor or a degradation product of the acylated peptide remains unclear [22]. Soares et al. proposed an alternative explanation for the production of nonacylated form, implicating that des-acyl ghrelin is a result of an incomplete acylation of ghrelin [23]. Des-acyl ghrelin and the acylated form share many nonendocrine actions, including the stimulation of food intake, modulation of cell proliferation, and minor effects on adipogenesis. However, it is still unclear that where the des-acylated form binds [18]. Baldanzi et al. suggested that there might be another ghrelin receptor distinct from GHSR type 1a (GHSR1a) [24]. Consequently findings identified that des-acyl ghrelin has specific receptors. Ghrelin is predominantly expressed in the stomach. Besides, it can be produced in significantly lower amount in several other tissues, such as ovary, placenta, kidney, pituitary, gland and pancreas [14, 18, 25–27]. In addition, ghrelin gene is also expressed in the central nervous system (CNS), including the inter-nuclear space between the lateral hypothalamus, the arcuate nucleus (ARH), the ventromedial nucleus (VMN), the dorsomedial nucleus (DMN), the paraventricular nucleus (PVN) and the ependymallayer of the third ventricle [28–30]. Furthermore, circulating ghrelin gains access to the CNS and reached different structures as hippocampus and ventral tegmental area (VTA) [30].

The GHS-R is a seven transmembrane-spanning domain G-protein coupled receptor that activates phospholipase C (PLC) [31]. PLC can consequently increase the intracellular Ca^2+^ levels through inositol-3-phopshate (IP3) and protein kinase C (PKC)-dependent pathways. There are two forms of ghrelin receptor, the GHS-R1a and GHS-R1b. The first one encodes for the full length biologically active receptor. The last one is a truncated isoform, which is therefore thought to represent a non-functional receptor form failing to bind ghrelin [32].

### Ghrelin and epilepsy: human studies

Recently, there is an increase in the number of human studies investigating the link between ghrelin and epilepsy. However, these findings are rather controversial. Aydin et al. first measured serum ghrelin levels in a immediate postictal period and found a lower ghrelin levels in serum compared with the healthy subjects, and the ghrelin levels increased with time [33]. They came up with explanations for the acute reduce of serum ghrelin levels. First, the reduction of serum ghrelin is due to a consumption to prevent the production of free radicals during a seizure. In addition, more ghrelin were taken in the CNS structures from peripheral system to counteract sepileptic discharges. These are in accordance with ghrelin's antiepileptic property. The other explanation is that ghrelin is decreased by a hormones feedback mechanism [34]. However, we think the hormones feedback mechanism might not explain the rapid changes within several hours because it needs a longer duration.

In the earlier study, the researchers detected that serum total ghrelin levels were higher in patients than in controls [35]. The researchers further demonstrated that this enhancement of serum ghrelin might promote the slow-way sleep and prolong non rapid eye movements sleep during the night, in which the seizures tend to occur [35]. However, many researchers discovered a decreased ghrelin levels in patients with epilepsy compared with the subjects in controls [36]. Suleyman et al. observed that serum and saliva ghrelin levels were significantly lower in epileptic patients than in controls before treatment, whereas they increased somewhat with treatment but still remained below the control values [33].

Another significant factor is the AEDs’ effect on ghrelin. Especially valproic acid (VPA), one of the most common AEDs, affect ghrelin levels due to its side effect of weight gain [37]. Most current epilepsy clinical studies don't encompass drug-naive group due to the ethical constrains and ghrelin levels were measured after several months of AEDs treatments. Reported findings of ghrelin levels in human studies are controversial. An increase of ghrelin levels were found in prepubertal epilepsy patients with VPA treatment [33, 38]. In contrast, a lower ghrelin levels during VPA treatment in adult and prepubertal subjects were also reported [39–41]. It's widely reported that ghrelin levels is decreased in obesity people and stimulated in patients with anorexia nervosa. Therefore, the initially VPA treatment may induce weight gain via increasing ghrelin levels [38] and the subsequent reduction of ghrelin might be due to the endocrine feedback regulation, for example the increased insulin or leptin [41]. Whereas the link between ghrelin and AEDs-induced weight changes still obscure. A recent literature reported that ghrelin levels were not significant changed in topiramate-induced weight loss patients [42].

The two major forms of ghrelin, acylated ghrelin and des-acyl ghrelin, were detected in serum, urine, and saliva of patients with pediatric epilepsy [43]. Before AEDs treatments, the acylated ghrelin levels were higher. The similar results are also detected in des-acyl ghrelin of urine and saliva, indicating that two different forms have similar regulating effect. Due to the neuroprotective and antiepileptic effects of ghrelin, ghrelin secretion is enhanced during the process of central lesions induced by seizures [19, 44–48].

Multiple variables are present in these studies as reviewed above. Age of patients with epilepsy, the type of epilepsy, the blood collection time and AEDs therapy all can affect the ghrelin levels, especially the AEDs’ effect on ghrelin. The basic consensus is that ghrelin levels have a tendency to decrease following epileptic episodes [41]. Further clinical studies are needed.

### Ghrelin and epilepsy: animal studies

As summarized in Table 1, several studies have confirmed that ghrelin has anticonvulsant properties. Obay et al. noted that intraperitoneal injections of ghrelin greatly delayed or prevented the development of pentylenetetrazole (PTZ)-induced epileptic seizures in rats [13]. The same group consequently reported that ghrelin can protect neurons by inhibiting the oxidative stress and preventing the decrease in antioxidant enzyme activities [49]. This may be a possible mechanism of ghrelin's anticonvulsant effect.

**Table 1:** Overiew of ghrelin's antiepileptic effects in different experimental models

Seizure model	Mechanisms	Main findings	Ref
**PTZ(rats)**		suppresses the onset time of PTZ-induced seizures.	[13]
**PTZ(rats)**	neuroprotection	inhibit lipid peroxidation, prevent reduction of antioxidant enzyme activities and GSH levels against PTZ-induced oxidative stress in a dose dependent manner.	[49]
**Pilocarpine(rats)**	neuroprotection	attenuate neuronal loss in hippocampus via activating PI3K/Akt pathways, reverse the decreased ratio of Bcl-2 to Bax,	[53]
**Pilocarpine (rats)**		Hexarelin and EP-80317, synthetic ligands of GHSR1a, were both able to prevent progression to status epilepticus	[54]
**KA(rats)**		Des-acyl ghrelin prolong the latency to status epilepticus.	
**Pilocarpine (GHSR−/− mice)**		increase the seizure threshold than their wild-type littermates.	[51]
***In vitro* models**		desensitization/internalization of the GHSR attenuate the epileptiform activity in hippocampal slices.	
**Pilocarpine (rats)**	neuroprotection	inhibite pilocarpine-induced apoptosis in hippocampus. AG increase the Bcl-2/Bax ratio and inhibited caspase-3 activation, increase the expression of phosphor-Akt, decrease the expression of phosphor-JNK in pyramidal neurons of hippocampus	[55]
**PTZ(rats)**	Improve spatial memory	intrahippocampal injection of ghrelin improve spatial memory.	[56]
**Pilocarpine**		des-acyl ghrelin significantly elevated seizure thresholds in C57Bl/6 and wild type mice but not in ghrelin receptor knock-out mice.	[52]
**PTZ(rats)**	Antiinflammation/neuroprotection	ghrelin administration reduce the levels of the pro-inflammatory cytokines TNF-α, IL-1β, and IL-6.reduces the levels of malondialdehyde and increases the serum levels of glutathione.	[57]

Lee et al. tried to block the anticonvulsant effect of ghrelin via an antagonist and found that the anticonvulsant effect of ghrelin on kainic acid (KA)-induced seizure activity was blocked by the ghrelin receptor antagonist D-Lys3-GHRP6 [50]. Consequently Portelli's group investigated that GHS-R deletion, inverse agonism, or desensitization leads to the attenuation of limbic seizures *in vivo* and epileptiform activity *in vitro*, which means that both agonists and inverse agonists for the GHS-R are capable of exerting anticonvulsant effects [51]. It has been found that the GHS-R knockout mice had a higher seizure threshold than their wild-type littermates when treated with pilocarpine. Des-acyl ghrelin was found to significantly elevate seizure thresholds in C57Bl/6 and wild type mice but not in ghrelin receptor knockout mice. It also has been found that des-acyl ghrelin exert anticonvulsant effect via ghrelin receptor rather not orexin pathway [52]. At present, there's still lack in studies investigating deep cellular and molecular mechanisms of ghrelin's antiepileptic effect. More studies need to be performed to investigate how ghrelin inhibit seizures.

### Ghrelin and epilepsy: regulation and functions in brain

### Ghrelin's role in hippocampal synaptic plasticity

Cognitive dysfunction is the most common complications of epilepsy [58]. The pathogenetic factors of cognitive impairment include seizures-related pathological changes, the duration and frequency of seizure, AEDs’ side effects and social psychological barriers [12]. Patients with temporal lobe epilepsy (TLE) are always complaining about their degeneration of memory [59]. Repeated attack of seizures cause lesions in hippocampus, a brain region playing a major role in memory formation [60]. In addition, patients with a history of epilepsy are found to have hippocampal atrophy [61–63]. Ghrelin appears to be related to memory retention and formation [64]. It's not hard to deduce that ghrelin may exert an effect as a palliative treatment for the memory impairments in epilepsy.

Hippocampus synaptic plasticity changes, including long-term potentiation (LTP), long-term depression (LTD) and structural synaptic changes, play an important role in learning and memory process [65]. Various changes of synaptic plasticity in hippocampal network occurred in accompany with acute seizures attack and consequently caused memory impairment [66]. Carlini et al. found for the first time that ghrelin administration increased memory retention in the hippocampus [67]. The effects of ghrelin on memory retention in hippocampus can be reversed by the treatment with fluoxetine (selective serotonin reuptake inhibitor) [68]. Intrahippocampus administration of ghrelin triggered a long-lasting potentiation of excitatory postsynaptic potentials (EPSPs) and induced population spikes (PSs) in the dentate gyrus of rats which might enhance the memory formation [65]. The phosphoinositide 3-kinase (PI3K) signaling pathways is involved in ghrelin's modulation [69]. Carlini et al. reported that ghrelin reduced the threshold for LTP generation via promoting the NO synthase (NOS) activity in dentate gyrus of rat hippocampus [70]. Moreover, ghrelin also was found to facilitate the dendritic spine synapse formation in hippocampus [70].

Ghrelin can increase delivery of α-amino-3-hydroxy-5-methyl-4-isoxazole propionic-type receptors (AMPARs) to synapses and produce functional modifications at excitatory synapses via activating GHS-R1a [71]. Ghrelin can promote the phosphorylation of GluN1 and amplified N-methyl-D-aspartate receptor (NMDAR)-mediated excitatory postsynaptic currents in the CA1 pyramidal cells of the hippocampus [72]. However, activation of AMPARs play a critical role in the generation of acute seizures and the antagonist of AMPARs, talampanel, is supposed to be an AED [73]. It's not coincident with ghrelin's antiepileptic actions. Further studies are needed to explain it.

A recent study demonstrated that microinjection of ghrelin into the CA1 region of hippocampus significantly improved the spatial learning and memory in PTZ-treated rats. [56]. There is still a lack of cellular and molecular mechanisms of ghrelin's memory promoting effect in epilepsy models. Although the anticonvulsant effect of ghrelin has been reported, whether the memory preservation effect involved in the antiepileptic mechanisms is still unclear. It can be certained that ghrelin can exert a protective actions from cognitive dysfunction in epileptogenesis by regulating hippocampal synaptic plasticity. Ghrelin may be used as a palliative treatment for the memory impairments in epilepsy.

### Modulatory effects on central neurotransmission and neuroendocrine

Abnormal excessive and synchronous neuronal activity in specific region of brain cause seizures [74]. Neuropeptide Y (NPY) and the inhibitory transmitter GABA exert inhibitory effects on seizures, whereas the excitatory transmitter glutamate facilitate the formation of seizures [75]. The imbalance of GABA and glutamate cause abnormal discharge, which facilitate the formation of seizures. Axons of hypothalamic ghrelin neurons abut NPY axons presynaptically in the ARH and in the paraventricular nucleus (PVH). Several studies have reported the ghrelin-NPY/GABA interactions in hypothalamic circuitry [8]. Ghrelin administration can promote the release of GABA in hypothalamic while the effects were not observed in extra-hypothalamus regions. So whether ghrelin's anticonvulsant effect is related to its modulation of neurotransmission needs to be further investigated.

Neuropeptides can also modulate seizures and epilepsy by regulating the release of traditional excitory/inhibitory transmitter. NPY is the most studied neuropeptide that is supposed to have anticonvulsant efficacy. Exogenous administration of NPY can suppress limbic seizure activity induced by KA [76]. Ghrelin has an effect on neuroendocrine system and alter the synthesis and release of neuropeptides. Ghrelin can promote the secretion of NYP by both neurotransmitter signaling pathways and endocrine secretion. Adrenocorticotopic hormone (ACTH) is used in treatment for infantile spasms, one of the intractable types of epilepsy occurs in infancy [77]. The stress/corticotrophin-releasing hormone (CRH) is reported to induce seizures in animal studies [78]. ACTH exerts its anticonvulsant effect via reducing the secretion of endogenous CRH [79]. It was well known that ghrelin has an stimulatory effect on the activity of hpothalamic-ptuitary-drenal (HPA) axis [80–82]. In healthy humans, ghrelin administration increases ACTH and cortisol plasma concentrations [83].

### Neuroprotective effect of ghrelin

A large number of data have shown that seizures can cause permanent damage to the CNS [84]. Abnormal electrical activity can cause extensive neuronal death or loss and network dysfunction in epilepsy-related brain regions, especially in hippocampus, amygdala and cortex [85]. Acute seizures can result in microvascular spasm and expose the nervous tissues to ischemia and hypoxia. The subsequently increased secretion of inflammation factor and excitatory amino acid may induce apoptosis via activating caspase protein family [86–88]. Hippocampal sclerosis and hyperplasia of astrocytes and microglias induced by neuronal damage were often observed in epileptic patients and models [89]. Several compounds with promising antiepileptic and neuroprotective properties were considered as new AEDs [90]. In recent studies, ghrelin was found to exert neuroprotective effects both peripherally and centrally, which might be a explanation for ghrelin's antiepileptic property [8].

Several studies have shown that ghrelin could stimulate proliferation and inhibit cell apoptosis in different cell types, including cardiomyocytes, aortic endothelial cells, ovarian follicle cells [91]. Recently, ghrelin was found to exert a similar effect in CNS. A decrease of the number of GABAergic interneurons were found in the dentate gyrus of TLE patients and rodent models [92]. Ghrelin can reduce the GABA loss within several hours after KA-induced acute seizures in hippocampus. The resulting reduction of inhibition lower the threshold of seizures [93]. Ghrelin was found to significantly reduce mitochondrial cytochrome c release, inhibit the activation of caspase-3 and increase Bcl-2/Bax ratio via activating the extracellular signal-regulated kinase (ERK)1/2 and PI3K/Akt signaling pathways [94–96]. More and more studies proved that ghrelin can function as a neuroprotective agent by inhibiting apoptotic pathways. In pilocarpine-induced seizures rats, ghrelin can significantly attenuate neuronal loss in hippocampal CA1 and CA3 regions via PI3K/Akt signaling pathways [53]. It's well known that oxidative stress is thought to play a role in the pathophysiology of epilepsy [97]. Ghrelin can attenuate oxidative stress-induced cell dysfunction *in vitro* [98]. In PTZ-induced seizure model, ghrelin can exert protective effects against oxidative damage and reduce neuronal death via diminishing oxidative stress and preventing the decrease in antioxidant enzyme activities [49, 57].

Hyperexcitability and inflammation induced by eastrocytes and microglias have a effect on epilepsy. Glia proliferation is charactered with sclerosis in epileptic foci, such as in hippocampus and temporal cortex [99]. Glia cells can secrete inflammatory cytokines. There is a positive feedback cycle between epileptogenesis and brain inflammation [100]. Spontaneous and recurrent seizures can promote the secretion of proinflammatory factor and inflammatory processes [101]. In addition, neuroinflammation also contributes to secondary seizures [102]. Lee et al. found that ghrelin can prevent KA-induced activation of microglia and astrocytes, and reduce the expression of proinflammatory mediators tumor necrosis factor alpha (TNF-α), interleukin-1beta (IL-1β), and cyclooxygenase-2 (COX-2) in hippocampus [103]. These results illuminate that ghrelin's antiepileptic effect is related to this neuroprotection and anti-inflammation efficacy.

### Ghrelin and hippocampal neurogenesis

Neurogenesis, a process of generating functionally integrated neurons from neuroblasts, involves proliferation, migration and differentiation of neuroblasts and the establishment of new circuitries [93, 104]. Several studies paid attention to the link between epilepsy and hippocampal neurogenesis. Dramatic increase of neurogenesis was reported in brains of patients and animal models with TLE at early stages after acute seizures [105]. The cell damage induced by acute seizures enhance the expression of neurotrophic factors in injury surrounding tissues. The elevated secretion of brain-derived neurotrophic factor (BDNF), vascular endothelial growth factor (VEGF), and others can indirectly cause neural stem cells (NSCs) proliferation [106]. However, hippocampus neurogenesis is evident to reduce at chronic stages in human TLE patients and in animal models of chronic TLE [107]. The decrease in neurogenesis, although not directly causes the occurrence of epilepsy, but may reduce the threshold of seizure threshold. Also, hippocampus neurogenesis is significantly relevant with the hippocampus-dependent learning and memory, which was often damaged during the process of epileptogenesis. Morphological and connectivity abnormalities of seizure-generated neurons are observed in most rodent models of mesial temporal lobe epilepsy (mTLE), including the extension of hilar basal dendrites and the ectopic migration of newborn granule cells into the polymorphic cell layer. Recent studies showed that the mammalian target of rapamycin (mTOR) signaling plays a significant role in the aberrant migration of newborn granule cells [108]. The cellular and molecular mechanisms of seizure-induced aberrant network reorganization is still unclear.

There are two hypotheses about the association of epilepsy and hippocampal neurogenesis. The first is that seizure-induced aberrant neurogenesis may contribute to the epileptic disease process. The other is that altered neurogenesis after seizures may represent an attempt of the injured brain to repair itself [106]. The reduction of seizure-generated neurons impairs epileptogenesis and reduces the frequency of spontaneous recurrent seizures [109, 110]. There is also evidence that seizure-induced neurogenesis may play a compensatory role in status epilepticus (SE) models using electrical stimulation to induce SE [111].

It has been shown that ghrelin increases cellular proliferation of adult rat hippocampal progenitor cells *in vitro* [112]. Peripheral administration of ghrelin stimulated neurogenesis in the dentate subgranular zone (SGZ) of adult male mice [113]. Ghrelin receptor knockout mice (Ghsr1a−/−) reduced numbers of progenitor cells in the dentate gyrus of the hippocampus and the systemic administration of ghrelin can reverse the decrement [114]. Ghrelin can promote the proliferation of hippocampal neural stem cell (NSC)s via activating the MEK/ERK1/2 and PI3K/Akt pathways [115]. Kent et al. demonstrated that systemic administration of physiological levels of acylated ghrelin for 8–10 days enhances hippocampal neurogenesis and spatial memory in rats [116]. Although the specific role of ghrelin-stimulated hippocampal neurogenesis plays in epiletological neurophysiology is still unclear. Precisely, ghrelin act as an effective factor for the maintenance of cognitive function during the progess of epileptogenesis.

### Acylated and des-acylated ghrelin

As mentioned above, des-acylated ghrelin represents approximately 80–90% of the total ghrelin detected in the serum. In the early stage of research, des-acylated ghrelin was considered to be devoid of biological effects [117, 118]. Subsequent research revealed that des-acylated ghrelin can affect food intake via the orexin pathway. Biagini et al. investigated that des-acylated ghrelin presented a trend in the prevention of pilocarpine-induced status epilepticus models [54]. Consistent with that, Portelli J found that des-acyl ghrelin can significantly elevated seizure thresholds and this action need ghrelin receptor to be involved [52]. Clinical studies tested des-acyl ghrelin levels in sersum and found that levels of acylated and des-acylated were in direct proportion to disease duration. More human studies are needed to investigate whether des-acylated or acylated ghrelin could be biomarkers in epileptogenesis progress and the deep mechanisms of des-acylated ghrelin's anticonvulsant efficacy.

### Neuropeptides vs traditional AEDs

Current available AEDs mainly exert therapeutic effects via targeting ion channels or ionotropic receptors to modulate intracellular second messenger, and thus inhibit abnormal exaltation [37]. It's well known that the traditional drugs have inferior binding affinity and potency for receptors, which cause brain functions injuries while exerting therapeutic effects [119]. Recently, more and more studies focus on the clinical application value of neuropeptides in neuronal disorders. Neuropeptides most are smaller and have a simpler structure than regular proteins [6]. Neuropeptide, also called as neuromodulator, can bind to their specific receptors to regulate postsynaptic cells to respond to the transmitters. Targeting neuropeptides system to treat epilepsy has more potential advantages, such as a higher binding affinity for their receptors and more security [7].

A number of endogenous peptides were reported to suppress seizures in the brain, such as NPY, somatostain, and galanin [120]. The discovery of anticonvulsant neuropeptides offers the possibility of new AEDs development. In addition, the changes of neuropeptides levels in serum are relevant with the duration and severity of epilepsy. Neuropeptides are also considered as biomarkers for clinical diagnose.

Whether the antiepileptic effect of ghrelin can be used into clinical application is still unknown. Ghrelin can directly cross the blood brain barrier (BBB) to convey its effect in brain [121]. This is the prerequisite for ghrelin to put into application. The interactions of epilepsy and antiepileptic medications on neuroendocrine system are so far not disclosed clearly. Neuropeptides can be new targets for the development of anticonvulsant drugs.

## CONCLUSIONS

Ghrelin is well reported to stimulate growth hormone (GH) secretion and regulate feeding behavior. There is considerable evidence showing that ghrelin is involved in regulation of several other important neurophysiological processes, including neuroprotection, neurotransmission and neurogenesis. Recently, Ghrelin was found to play a major role in inhibiting seizures. In this article, we reviewed the recent evidence of ghrelin’ potential antiepileptic actions and its possible mechanisms.

The results in clinical studies were controversial because several influencing factors exist. AEDs therapy and the age of patients both affect the ghrelin levels in serum. A general consensus is that the blood ghrelin levels have a declining trend in both experimental epileptic rodents and in patients. Although the results of the relating studies *in vitro* and *in vivo* are partially contradictory, it could be established that ghrelin has anticonvulsant properties. The potential mechanisms of ghrelin's anticonvulsant actions may be explained in the following three aspects: 1. Ghrelin can dampen cognitive dysfunction in epileptogenesis by promoting hippocampal synaptic plasticity. 2. Ghrelin exerts anticonvulsant efficacy via stimulating secretion of NPY/ACTH. Ghrelin's modulation of neurotransmission may involved in the anticonvulsant actions. 3.Ghrelin exerts neuroprotective and anti-inflammatory effects and protect neuron from epilepsy-induced damage.

We first present that the ghrelin-stimulated neurogenesis in hippocampus have significant effect in pathophysiology of epilepsy. The deep cellular and molecular mechanisms of ghrelin's antiepileptic effect is still obscure. Novel treatments targeting neuropeptides have more potential advantages in comparison to classic AEDs. Future studies are still needed to investigate the mechanism. The role of des-acyl ghrelin plays in epilepsy also need further studies.
